# Emergence of Non-Hemadsorbing African Swine Fever Virus Genotype II Variants and the Evolution of a Vaccine-Derived Strain in Vietnam

**DOI:** 10.3390/v18060606

**Published:** 2026-05-26

**Authors:** Thi Chau Giang Tran, Thi Tam Than, Thi Ngoc Ha Lai, Hoang Duc Le, Trong Tung Nguyen, Ngoc Duong Vu, Ngoc Bao Anh Ngo, Hoai Thuong Nguyen, Phuong Anh Nguyen, Kalhari Goonewardene, Aruna Ambagala, Van Phan Le

**Affiliations:** 1 College of Veterinary Medicine, Vietnam National University of Agriculture, Hanoi 100000, Vietnam; chaugiangtran1205@gmail.com (T.C.G.T.); duong.vnd99@gmail.com (N.D.V.); ngongocbaoanh@gmail.com (N.B.A.N.); thuong2211854@gmail.com (H.T.N.); phuonganhnguyen2601@gmail.com (P.A.N.); 2 Laboratory of Viral Infectious Diseases, Center for Research Excellence and Innovation, Vietnam National University of Agriculture, Hanoi 100000, Vietnam; thantam207@gmail.com; 3 Vettech Veterinary Joint Stock Company, Hanoi 100000, Vietnam; hoangocha1998@gmail.com; 4 Institute of Biology, Vietnam Academy of Science and Technology, Hanoi 100000, Vietnam; lehoangduc@ib.ac.vn; 5 Swine Viral Evolution and Vaccine Development Research Unit, Chulalongkorn University, Bangkok 10330, Thailand; ngttung.vet@gmail.com; 6 Canadian Food Inspection Agency, National Centre for Foreign Animal Disease, Winnipeg, MB R3E 3R2, Canada; kalhari.goonewardene@inspection.gc.ca

**Keywords:** African swine fever virus genotype II, non-hemadsorbing strain, vaccine-derived strain, porcine, Vietnam

## Abstract

Highly virulent African swine fever virus (ASFV) genotype II strains have been responsible for the global epidemic in domestic pigs and are typically characterized by a hemadsorption (HAD)-positive phenotype mediated by the CD2v protein encoded by the *EP402R* gene. Here, we report the detection and genetic characterization of three non-HAD genotype II ASFV isolates (VNUA/ASFV/VP2023-isolate1, VNUA/ASFV/TB2024-isolate2, and VNUA/ASFV/HY2024-isolate3) recovered from whole-blood samples collected from pigs exhibiting prolonged clinical signs in northern Vietnam. Whole-genome analysis revealed nonsense mutations in the *EP402R* gene (G57A in isolates VNUA/ASFV/TB2024-isolate2 and VNUA/ASFV/HY2024-isolate3, and G132A in VNUA/ASFV/VP2023-isolate1), resulting in premature stop codons and a HAD-negative phenotype. Furthermore, additional genetic alterations, including deletions and frameshift mutations, were identified within multigene families (*MGF110*, *MGF360*, and *MGF505*), which are known to play critical roles in virulence, host range, and immune evasion. Notably, VNUA/ASFV/VP2023-isolate1 harbored a partial deletion of the *I177L* gene along with the insertion of an *mCherry* marker gene, suggesting possible evolution of the modified live ASFV-G-Δ*I177L* vaccine strain under field conditions. Collectively, these findings underscore the ongoing evolution and genomic plasticity of ASFV strains circulating in Vietnam.

## 1. Introduction

African swine fever (ASF) is a devastating transboundary disease of domestic and wild pigs caused by the ASF virus (ASFV), leading to severe socioeconomic losses worldwide [1]. Mortality rates of ASF may reach 100%, depending on viral virulence and host factors. ASFV is a large, enveloped double-stranded DNA virus belonging to the family *Asfarviridae* [2]. Historically, all known ASFV strains have been classified into 24 p72 genotypes and eight serogroups based on sequence analysis and hemadsorption inhibition assays, respectively [3,4,5]. However, recent studies have refined these classifications, demonstrating that the previously designated genotype XVIII represented a mixed population of genotypes I and VIII [6], while recent seroimmunotyping analyses have expanded the number of recognized serogroups [7]. To date, only ASFV genotypes I and II have been reported outside Africa. Following the introduction of genotype II into Georgia in 2007, the virus spread extensively across Europe and Asia, reaching China in 2018 and Vietnam in 2019 [8,9]. Low-virulent ASFV genotype I strains causing chronic infection were reported in China in 2021 [10]. The co-circulation of genotype I and II strains in China has resulted in the emergence of highly virulent recombinant variants [11], which were subsequently detected in Vietnam in 2023 [12]. These developments underscore the ongoing evolution and expanding genetic complexity of ASFV in endemic regions.

ASFV genomes exhibit considerable variability, particularly within multigene family (MGF) regions and intergenic regions (IGRs), which play key roles in immune evasion, virulence modulation, and host adaptation [13,14,15,16]. Several other genes have also been directly implicated in virulence. For example, deletion of the *I177L* gene results in attenuation (ASFV-G-∆*I177L*) and has been utilized in the development of live attenuated vaccine candidates [17]. To enable differentiation of infected from vaccinated animals (DIVA), the *I177L* gene was replaced with the *mCherry* reporter gene, which allows for identification of the vaccine strain via fluorescence and PCR, while the absence of the *I177L* gene distinguishes it from the wild-type virus [17]. To enable serological differentiation of pigs vaccinated with the ASFV-G-∆I177L-based vaccine licensed in Vietnam [18], an experimental ASFV-G-∆I177L/∆EP402R double-deletion vaccine candidate was subsequently developed through deletion of the EP402R gene [19]. Deletion of the *EP402R* gene alone, however, from the highly virulent ASFV genotype II Georgia 2010 isolate did not attenuate the virus [20].

A hallmark of most highly virulent genotype II strains is their ability to induce hemadsorption (HAD) in infected porcine alveolar macrophages (PAMs), mediated by the CD2v protein encoded by *EP402R* [21,22]. Because of the strong association between the HAD-positive phenotype and most virulent genotype II strains, hemadsorption is widely used as a phenotypic marker in ASFV characterization. However, naturally occurring HAD-negative genotype II isolates have recently been reported in Europe and Asia [23,24]. Loss of hemadsorption has been linked to genetic alterations in *EP402R*, including point mutations and truncations that disrupt CD2v expression [23,24,25,26].

In Vietnam, several live attenuated ASF vaccines have been granted commercial licenses and are currently used in commercial pig production systems, including NAVET ASFVAC, which is derived from the ASFV-G-∆I177L strain, and AVAC ASF LIVE, which is derived from an ASFV-G-∆MGF strain. These vaccines are implemented under national vaccination programs coordinated by the Ministry of Agriculture and Rural Development, with post-vaccination monitoring involving assessment of clinical signs, herd mortality, and reporting of ASF outbreaks through provincial veterinary authorities [27]. The widespread use of live attenuated vaccines in endemic settings may impose additional evolutionary pressures on circulating ASFV populations and complicate molecular epidemiological investigations, particularly when vaccine-derived or recombinant viruses emerge in the field.

Despite increasing reports of HAD-negative ASFV genotype II isolates, their evolutionary origins, genomic diversity, and epidemiological significance remain poorly understood, largely due to the limited availability of complete genome sequences. This knowledge gap is particularly concerning in regions where live attenuated vaccines are under development or deployment, as genomic changes affecting virulence-associated genes or marker insertions may complicate diagnosis, surveillance, and differentiation between field and vaccine-derived strains. To address this urgent need, we performed whole-genome sequencing and comparative genomic analysis of three HAD-negative ASFV genotype II isolates detected in Vietnam. We analyzed their phylogenetic relationships and characterized genetic variations in the *EP402R*, *I177L*, and multigene family regions to elucidate the evolutionary dynamics and potential implications of these low-virulence, non-hemadsorbing strains.

## 2. Materials and Methods

**Figure 1:**
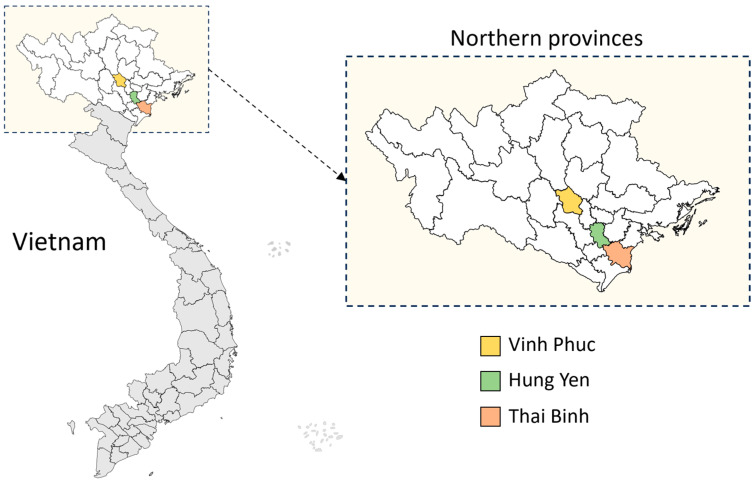
Geographic locations of the sampled provinces in Vietnam. Colored provinces (Vinh Phuc, Hung Yen, and Thai Binh) indicate the sampled locations. White areas represent other provinces in northern Vietnam, and grey areas represent provinces in central and southern Vietnam.

Three whole-blood samples originating from unvaccinated finishing pigs and sows from three different farms, exhibiting prolonged clinical signs consistent with ASF, including fever, anorexia, lethargy and low mortality rates at the time of sampling, were used in this study (Table 1). The samples were designated VNUA/ASFV/VP2023-isolate1, collected in Vinh Phuc Province in 2023, VNUA/ASFV/TB2024-isolate2, collected in 2024 in Thai Binh Province, and VNUA/ASFV/HY2024-isolate3, collected in Hung Yen Province in 2024. All three provinces are in northern Vietnam and are geographically proximate (Figure 1). This study follows the former provincial administrative structure of Vietnam, consisting of 63 provinces, as the samples were collected in 2023–2024, before the reorganization of provincial-level administrative subdivisions in July 2025 (reducing the number to 34 provinces).

Viral DNA was extracted from whole-blood samples using the Patho Gene-spin™ DNA/RNA Extraction Kit (iNtRON Biotechnology, Republic of Korea) according to the manufacturer’s instructions. ASFV genomic DNA was detected using the VDx^®^ ASFV qPCR Ver. 2.1 kit (Median Diagnostics, Seoul, Republic of Korea), followed by Phan G1 and G2 genotyping qPCR assays using 4× CAPITAL™ 1-Step qRT-PCR Probe Master Mix (Biotechrabbit, Berlin, Germany), as previously described [28]. All assays were performed on a CFX96 Touch Real-Time PCR Detection System (Bio-Rad Laboratories, Hercules, CA, USA).

Primary porcine alveolar macrophages (PAMs) were prepared from lung tissues obtained from healthy piglets originating from an ASF-negative farm. The donor animals were screened and confirmed negative for major swine pathogens, including ASFV, classical swine fever virus (CSFV), porcine reproductive and respiratory syndrome virus (PRRSV), and porcine circovirus type 2 (PCV2), following previously reported procedures [29]. After isolation, the PAM cells were washed thoroughly to eliminate residual tissue debris, suspended in RPMI 1640 medium (Gibco, Thermo Fisher Scientific, Grand Island, NY, USA) supplemented with 10% fetal bovine serum and antibiotic-antimycotic solution, and seeded into culture plates. The cultures were maintained at 37 °C in a humidified incubator containing 5% CO_2_ for 24 h to allow macrophage attachment before virus inoculation. The three whole-blood samples collected from ASFV-infected pigs described above in EDTA tubes were processed according to World Organization for Animal Health (WOAH) guidelines [30]. Clarified sample suspensions were inoculated onto PAM monolayers prepared in 6-well plates (Thermo Fisher Scientific, Grand Island, NY, USA) at a density of 1 × 10^6^ cells/mL. 100 µL of sample suspension in 2 mL of culture medium was added to each well, and the plates were subsequently incubated at 37 °C with 5% CO_2_. At 48 h post-infection, pig erythrocytes were added to a final concentration of 1% (*v*/*v*) to evaluate hemadsorption (HAD). HAD activity was assessed between 72 and 96 h post-infection by observing the formation of characteristic rosette- or star-shaped erythrocyte aggregates surrounding infected PAMs under a microscope. Viral replication in PAM cultures was confirmed by qPCR as described above, and the resulting ASFV isolates were subsequently used for whole-genome sequencing.

For each isolate, 100 µL of DNA extracted from the cell culture supernatant was submitted to Macrogen Inc. (Seoul, Republic of Korea) for whole-genome sequencing. Libraries were prepared using the Illumina TruSeq Nano DNA Library Prep Kit, and whole-genome sequencing was performed on an Illumina platform, generating 151 bp paired-end reads. Raw sequencing data were first assessed for quality using FastQC version 0.12.1 [31]. Adapter trimming and quality filtering were performed using fastp version 1.0.1 [32], with reads shorter than 50 bp removed, a minimum Phred quality score of 20 applied, and low-quality bases trimmed from both 5′ and 3′ ends. The filtered reads were then mapped to the host reference genome (Sus scrofa) using BWA-MEM2 version 2.2.1 [33] to remove host-derived sequences. Unmapped reads were extracted using SAMtools version 1.6 [34] and subjected to de novo assembly using SPAdes version 3.15.5 [35].

Assembly quality was evaluated using QUAST version 5.3.0 [36] by comparison with the ASFV Georgia 2007/1 reference genome [37]. The longest contig from each assembly was selected as the representative genome for downstream analyses. Sequencing depth was estimated as the total number of mapped bases divided by the reference genome size, calculated as the product of the number of reads mapped to the reference and the average read length [38,39]. In addition to mean sequencing depth, the range of per-position coverage values was calculated to describe coverage distribution across the reference genome. Genome annotation was performed by transferring open reading frames (ORFs) from ASFV Georgia 2007/1 using GATU [40], followed by manual curation in Geneious Prime version 2026.0.2. Multiple sequence alignments were generated using MAFFT version 7.525 [41] with the default FFT-NS-2 algorithm. Phylogenetic trees based on the p72 and p54 genes were reconstructed using the maximum likelihood method in MEGA X [42]. Whole-genome phylogenetic analysis was performed using IQ-TREE version 2.0.7 [43], with the best-fit substitution model selected based on the lowest Bayesian information criterion (BIC). Branch support was evaluated using 1000 bootstrap replicates, and trees were visualized using iTOL version 7.4.1 [44]. A circular genome comparison between ASFV Georgia 2007/1 and the three study isolates was generated using the CGView Comparison Tool version 2.0.3 [45].

## 3. Results

### 3.1. Detection of ASFV and Non-Hemadsorption Phenotype

All three field samples were confirmed positive for ASFV using the VDx^®^ ASFV qPCR Ver. 2.1 kit (Median Diagnostics, Seoul, Republic of Korea). The initial cycle threshold (Ct) values were 24.25 for VNUA/ASFV/VP2023-isolate1, 30.25 for VNUA/ASFV/TB2024-isolate2, and 29.83 for VNUA/ASFV/HY2024-isolate3, indicating moderate viral loads in whole-blood samples. Genotyping was performed using previously described Phan G1 and Phan G2 qPCR assays specific for ASFV genotypes I and II, respectively [28]. All three samples tested positive in the Phan G2 qPCR assay, which is specific for ASFV genotype II, but were negative in the Phan G1 assay.

**Figure 2:**
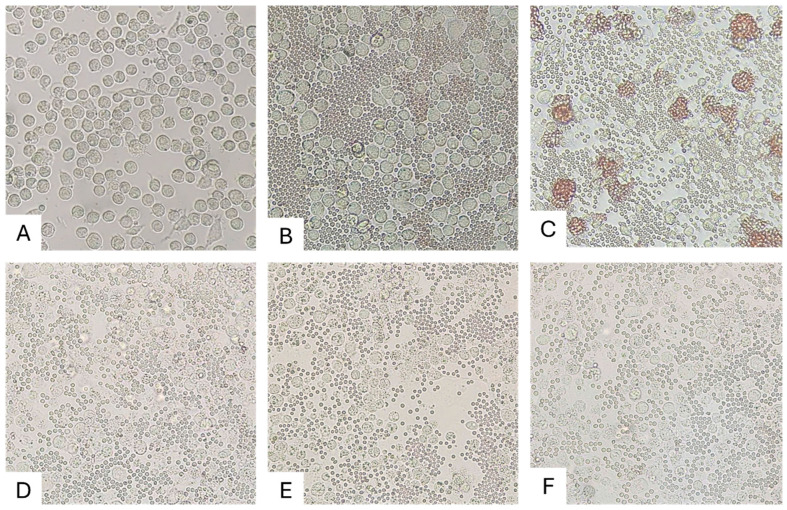
Hemadsorption (HAD) assay results for the three isolates obtained from whole blood samples collected from pigs showing prolonged clinical signs. (**A**) Uninoculated porcine alveolar macrophages (PAMs) without added red blood cells. (**B**) Uninoculated PAMs with added red blood cells. (**C**) Positive control: PAMs infected with VNUA/HY-ASF1 [46], demonstrating characteristic hemadsorption (HAD) rosette formation. (**D**–**F**) PAMs infected with VNUA/ASFV/VP2023-isolate1, VNUA/ASFV/TB2024-isolate2, and VNUA/ASFV/HY2024-isolate3, respectively, showing cytopathic effects (CPE) in the absence of HAD. Images were captured at 40× magnification.

Following inoculation into primary porcine alveolar macrophages (PAMs), all samples induced clear cytopathic effects (CPE), but no hemadsorption was observed (Figure 2). In contrast, the positive control strain (VNUA/HY-ASF1) exhibited characteristic rosette formation surrounding infected cells. Viral replication in cell cultures was confirmed by qPCR, with Ct values decreasing to 18.53, 19.47, and 19.90 for VNUA/ASFV/VP2023-isolate1, VNUA/ASFV/TB2024-isolate2, and VNUA/ASFV/HY2024-isolate3, respectively.

### 3.2. Whole Genome Sequencing and Assembly Metrics

High-throughput Illumina sequencing generated between 33.9 and 46.9 million paired-end reads per sample (150 bp read length). After quality filtering and removal of host-derived reads, 0.30–1.02% of the reads mapped to the ASFV genome. De novo assembly using SPAdes yielded a single dominant contig for each isolate. The assembled genome lengths were 186,729 bp, 183,798 bp, and 183,745 bp for VNUA/ASFV/VP2023-isolate1, VNUA/ASFV/TB2024-isolate2, and VNUA/ASFV/HY2024/isolate3, respectively. The GC content was highly conserved across the isolates (38.5–38.6%). Mean sequencing depth ranged from 85× to 369× (Table 1), with per-position coverage ranging from 100× to 1417× for VNUA/ASFV/VP2023-isolate1, 116× to 1026× for VNUA/ASFV/TB2024-isolate2, and 32× to 364× for VNUA/ASFV/HY2024-isolate3, providing strong support for base-level accuracy. QUAST analysis confirmed high assembly quality, with genome coverage ranging from 96.4% to 97.4% relative to the ASFV Georgia 2007/1 reference strain, and no major structural misassemblies were detected.

The slightly shorter genome lengths compared with the 190,584 bp Georgia 2007/1 reference genome were primarily attributable to incomplete recovery of the terminal inverted repeat (TIR) regions, a known limitation of short-read Illumina sequencing for large double-stranded DNA viruses. Genome annotations identified 183, 179, and 179 predicted open reading frames (ORFs) in VNUA/ASFV/VP2023-isolate1, VNUA/ASFV/TB2024-isolate2, and VNUA/ASFV/HY2024-isolate3, respectively.

### 3.3. Genomic Similarity and Phylogenetic Relationships

Pairwise nucleotide identity analysis demonstrated that the VNUA/ASFV/TB2024-isolate2 and the VNUA/ASFV/HY2024-isolate3 were nearly identical (99.99%). In contrast, VNUA/ASFV/VP2023-isolate1 exhibited greater divergence, sharing only 97.67% nucleotide identity with the other two isolates. When compared with representative genotype II reference strains, including Georgia 2007/1, China/2018/AnhuiCGQ [47], and the Vietnamese field strain VN/HY-ASFV1 [46], nucleotide identities ranged from 98.68% to 98.97%. Notably, VNUA/ASFV/VP2023-isolate1 showed slightly higher sequence similarity to these reference strains than did VNUA/ASFV/TB2024-isolate2 and VNUA/ASFV/HY2024-isolate3.

**Figure 3:**
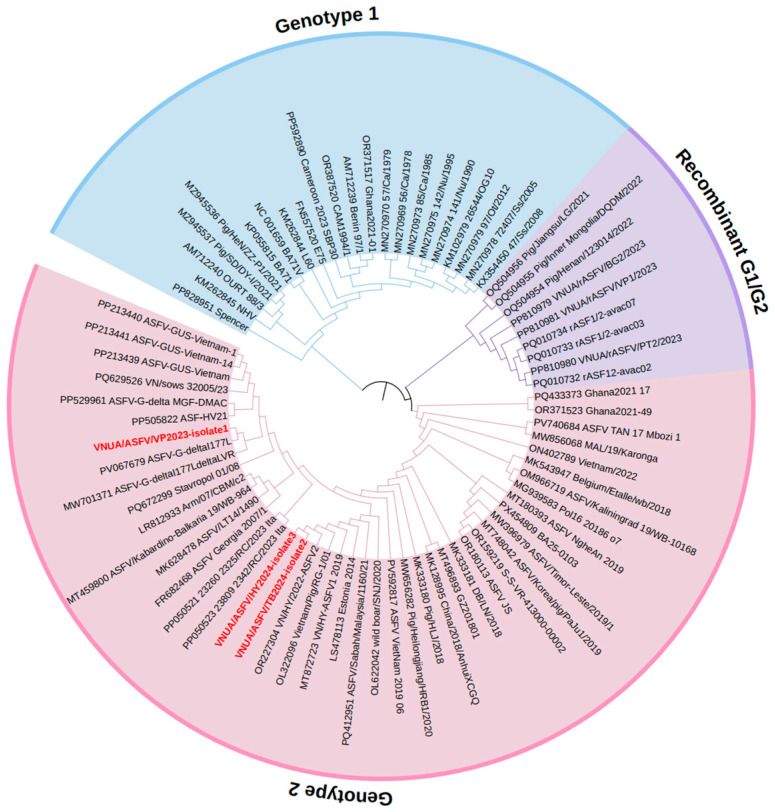
Maximum likelihood phylogenetic tree based on whole-genome sequences of ASFV strains. The tree was generated using IQ-TREE (1000 bootstrap replicates) and visualized with iTOL Version 7.4.1 (Interactive Tree of Life, https://itol.embl.de (accessed on 26 January 2026). The three isolates from this study are shown in bold red.

Phylogenetic analysis based on whole-genome sequences of ASFV genotype I, genotype II, and recombinant genotype I/II strains demonstrated that VNUA/ASFV/TB2024-isolate2 and VNUA/ASFV/HY2024-isolate3 clustered with contemporary Vietnamese field isolates of ASFV genotype II, whereas VNUA/ASFV/VP2023-isolate1 was more closely related to *I177L*-deleted vaccine-derived strains, including ASFV-G-Δ*I177L* and ASFV-G-Δ*I177L*/ΔLVR [48] (Figure 3).

### 3.4. Intergenic Region (IGR) Typing

**Figure 4:**
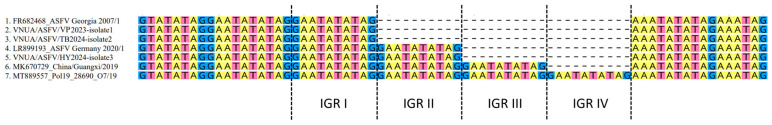
Multiple sequence alignment of the *I73R*–*I329L* intergenic region of the three isolates and reference strains belongs to IGR I to IV variants. Dashed vertical lines indicate distinct repeat blocks.

To further characterize the three non-HAD isolates from this study, the intergenic region between *I73R* and *I329L* of the three isolates was analyzed. It was revealed that the VNUA/ASFV/VP2023-isolate1 and VNUA/ASFV/TB2024-isolate2 are IGR I variants, whereas VNUA/ASFV/HY2024-isolate3 is an IGR II variant (Figure 4).

### 3.5. Genome-Wide Comparative Analysis and Mutational Landscape

**Figure 5:**
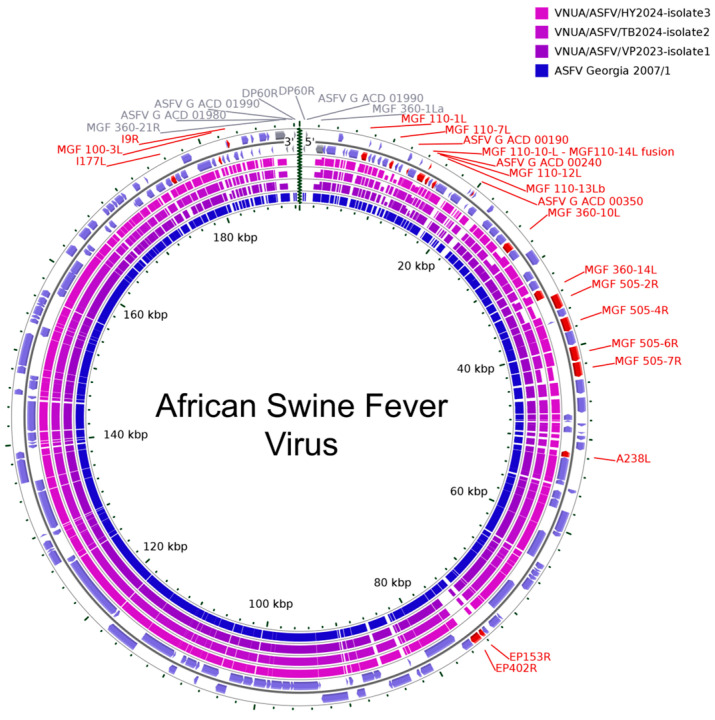
Circular plot comparing the genomes of ASFV Georgia 2007/1 and the three isolates from this study based on ORF-level similarity. Each concentric ring represents a viral genome, with ORFs displayed according to their genomic positions. Gray-labeled ORFs indicate coding regions that are expected to be located within the missing terminal regions at the 5′ and/or 3′ ends of the genomes belonging to the three isolates. Red-labeled ORFs denote genes harboring major mutations, including large deletions, frameshift mutations, or premature stop codons, relative to the reference genome. The height of each ORF segment is proportional to its nucleotide identity percentage compared with ASFV Georgia 2007/1.

The assembled genomes of the three isolates from this study were 3855–6839 bp shorter than that of the reference genome of the Georgia 2007/1 strain (Figure 5). This reduction was primarily attributable to incomplete recovery of terminal genomic regions, a known limitation of short-read Illumina sequencing due to terminal inverted repeats (TIRs) and repetitive structures. Specifically, VNUA/ASFV/VP2023-isolate1 lacked 2252 bp at the 5′ end and 1962 bp at the 3′ end; VNUA/ASFV/TB2024-isolate2 lacked 2348 bp and 2058 bp; and VNUA/ASFV/HY2024-isolate3 lacked 2380 bp and 2090 bp at the 5′ and 3′ ends, respectively. These terminal regions include several ORFs such as *DP60R*, *ACD_01990*, *ACD_01980*, and partial sequences of *MGF360-1La* and *MGF360-21R*. The genome of VNUA/ASFV/VP2023-isolate1 was longer than the other two isolates due to the insertion of the mCherry reporter cassette into the genome.

To systematically characterize genomic variation, mutations were classified into three categories: (i) major deletions (MD), defined as deletions resulting in partial or complete loss of ORFs; (ii) frameshift mutations (FM), caused by nucleotide insertions or deletions disrupting the reading frame; and (iii) nonsense mutations (NM), defined as single-nucleotide substitutions introducing premature stop codons. Most mutations were located within the multigene family (MGF) 110, 360, and 505 regions (Table 2). Overall, VNUA/ASFV/TB2024-isolate2 and VNUA/ASFV/HY2024-isolate3 exhibited highly similar mutational profiles, whereas VNUA/ASFV/VP2023-isolate1 harbored fewer MGF-associated disruptions.

#### 3.5.1. Major Deletions

Large deletions were predominantly observed within the MGF110 and MGF505 regions. In the MGF110 cluster, a 652 bp deletion (positions 10,177–10,829 relative to Georgia 2007/1) resulted in the complete loss of the *MGF110-7L* gene in VNUA/ASFV/TB2024-isolate2 and VNUA/ASFV/HY2024-isolate3. In contrast, VNUA/ASFV/VP2023-isolate1 contained a distinct 708 bp deletion (positions 14,316–15,024) that partially removed the *MGF110-10L–MGF110-14L* fusion gene, completely deleted *ASFV G ACD_00240*, and partially deleted *MGF110-12L*. Within the MGF505 cluster, a 1704 bp deletion (positions 41,199–42,903) was identified in VNUA/ASFV/TB2024-isolate2 and VNUA/ASFV/HY2024-isolate3. This deletion removed the terminal 394 bp of *MGF505-6R* and the initial 1181 bp of *MGF505-7R*, generating a fusion ORF from the remaining gene fragments. Additionally, *MGF100-3L* in VNUA/ASFV/VP2023-isolate1 harbored a 33-nucleotide deletion (positions 58–90).

#### 3.5.2. Frameshift Mutations

Frameshift mutations were the most prevalent genomic alterations. In VNUA/ASFV/VP2023-isolate1, a single-nucleotide insertion (G) between positions 641 and 642 of *MGF110-1L* extended the ORF from 645 bp to 810 bp. In VNUA/ASFV/TB2024-isolate2 and VNUA/ASFV/HY2024-isolate3, deletions within poly-C tracts in the *MGF110-10L–MGF110-14L* fusion gene introduced premature stop codons, shortening the ORF from 819 bp to 345 bp and 366 bp, respectively.

All three isolates exhibited deletions within a poly-C region of *MGF110-13Lb* (8 of 17 nucleotides removed), producing slightly elongated ORFs (381 bp versus 369 bp in the reference). A poly-G tract mutation in *ASFV G ACD_00350*, where deletions of 3, 8, and 9 G, resulted in truncated ORFs of 129 bp (isolate1), 42 bp (isolate2), and 69 bp (isolate3), compared with 132 bp in the reference. Sequence data obtained for the homopolymeric regions were supported by high sequencing depth across the affected loci, ranging from 54× to 220× for the *MGF110-10L–MGF110-14L* fusion gene, 62× to 410× for *MGF110-13Lb*, and 51× to 259× for ASFV_G_ACD_00350, confirming the reliability of the observed indels despite the known sequencing challenges associated with poly-C/G tracts.

Additional frameshift mutations were detected in the MGF360 and MGF505 genes of VNUA/ASFV/TB2024-isolate2 and VNUA/ASFV/HY2024-isolate3. A single-nucleotide (C) deletion at position 451 of *MGF360-10L* truncated the ORF from 1038 bp to 456 bp. A nucleotide (C) insertion between positions 850 and 851 of *MGF360-14L* shortened the ORF from 1074 bp to 864 bp. In *MGF505-2R*, a two-nucleotide (C and T) deletion reduced the ORF from 1581 bp to 786 bp. A single-nucleotide insertion in *MGF505-4R* was detected only in VNUA/ASFV/HY2024-isolate3, producing a truncated ORF of 1482 bp.

Among non-MGF genes, *A238L* in VNUA/ASFV/TB2024-isolate2 and VNUA/ASFV/HY2024-isolate3 contained a single-nucleotide (G) insertion, resulting in ORF elongation (717 bp compared with 690 bp in the Georgia 2007/1 reference strain). *EP153R* exhibited frameshift mutations in all three isolates, caused by a single-nucleotide (A) deletion at position 52 in VNUA/ASFV/TB2024-isolate2 and VNUA/ASFV/HY2024-isolate3, or a single-nucleotide (C) insertion between positions 188 and 189 in VNUA/ASFV/VP2023-isolate1. These mutations led to truncated ORFs of 63 bp (VNUA/ASFV/TB2024-isolate2 and VNUA/ASFV/HY2024-isolate3) and 195 bp (VNUA/ASFV/VP2023-isolate1), compared with the 477 bp ORF in the Georgia 2007/1 reference strain. Additional disruptions were observed in *I9R* (elongation to 312 bp) and *ASFV-G ACD_00190* (truncation to 75 bp).

#### 3.5.3. Nonsense Mutations and Disruption of EP402R

**Figure 6:**
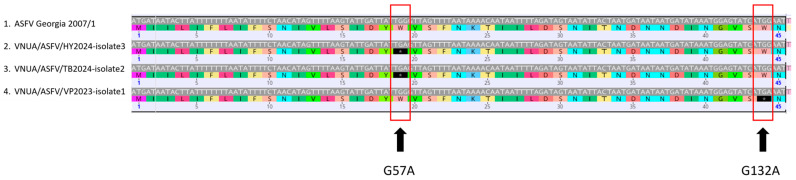
Disruption of the *EP402R* (CD2v) coding sequence by premature stop codons. Black boxes with asterisks indicate positions containing premature stop codons.

A nonsense mutation in *MGF110-1L* was identified in VNUA/ASFV/TB2024-isolate2 and VNUA/ASFV/HY2024-isolate3 (C590T), shortening the ORF from 645 bp to 591 bp. Most notably, analysis of the *EP402R* gene revealed two distinct nonsense mutations (Figure 6). A G57A substitution was detected in VNUA/ASFV/TB2024-isolate2 and VNUA/ASFV/HY2024-isolate3, whereas a G132A substitution was observed in VNUA/ASFV/VP2023-isolate1. Both mutations introduced premature stop codons near the 5′ region of the 1083 bp *EP402R* gene, abolishing translation of a full-length CD2v protein. This genetic disruption is consistent with the hemadsorption-negative (HAD−) phenotype observed in vitro.

#### 3.5.4. Disruption of the *I177L* Locus in VNUA/ASFV/VP2023-Isolate1

**Figure 7:**
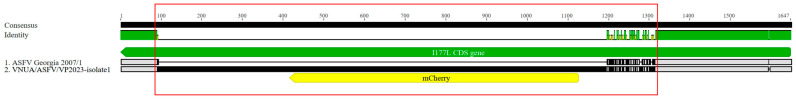
Sequence alignment of the modified *I177L* locus in VNUA/ASFV/VP2023-isolate1 against a reference ASFV Georgia 2007/1. Grey regions represent aligned sequence regions shared between the compared ASFV strains, black regions indicate unmatched sequence regions.

Extensive disruption of the *I177L* gene was observed in VNUA/ASFV/VP2023-isolate1. Truncation of the coding sequence, together with a large insertion (mCherry reporter gene), resulted in an expanded locus exceeding the 1112 bp length of the ASFV Georgia 2007/1 reference genome (Figure 7). Comparative genomic analysis revealed 99.6% nucleotide identity with the reported ASFV-G-Δ*I177L* strain and lower identity (93.8%) with ASFV-G-Δ*I177L*/ΔLVR, another vaccine candidate strain licensed in Vietnam. The reduced similarity to ASFV-G-Δ*I177L*/ΔLVR was primarily attributable to a large 10,861 bp deletion encompassing multiple MGF genes in that strain, which was absent in VNUA/ASFV/VP2023-isolate1. Notably, additional deletions involving the *MGF110-10L–MGF110-14L* region, *ASFV G ACD_00240*, *MGF110-12L*, and *MGF100-3L*, as well as nonsense mutations in *EP153R* and *EP402R*, were unique to VNUA/ASFV/VP2023-isolate1 and were not detected in the vaccine strains.

The mutations identified within the *EP153R* and *EP402R* genes were supported by high-quality reads with regional sequencing coverage ranging from 46× to 386×, confirming that these genetic alterations represent genuine evolutionary events rather than sequencing artifacts caused by read dropouts.

## 4. Discussion

In Vietnam, ASFV strains are becoming increasingly heterogeneous, with multiple strains exhibiting distinct pathogenic profiles co-circulating in pig populations. These include historic, highly pathogenic p72 genotype II strains and recently emerged recombinant p72 genotype I/II strains associated with acute disease and vaccine-like genotype II strains linked to chronic clinical manifestations. The growing genetic diversity of ASFV in the field has raised important concerns regarding viral evolution, potential changes in virulence, and genetic recombination [49]. Experimental evidence further indicates that closely related ASFV strains can undergo frequent recombination under both in vitro and in vivo conditions, generating genetically diverse progeny and accelerating viral evolution [50]. In this context, whole-genome sequencing and detailed molecular characterization of circulating ASFV strains are essential for identifying genetic changes that may influence viral phenotype, transmission dynamics, and control strategies.

In the present study, three ASFV genotype II isolates were successfully isolated from pigs exhibiting prolonged clinical signs. All three isolates displayed a non-hemadsorbing (HAD−) phenotype in contrast to HAD-positive classical ASFV genotype II and recombinant genotype I/II strains reported to date from Vietnam. HAD activity is mediated by the CD2v protein encoded by the *EP402R* gene and is generally associated with virulent field strains [23,51]. Consistent with this phenotype, whole-genome analysis identified two nonsense mutations (G57A in 2024 and G132A in 2023) in the *EP402R* gene, both introducing premature stop codons near the 5′ region. A recent publication also reported the detection of ASFV strains containing the G57A mutation in four field samples collected from southern and central provinces of Vietnam in 2023 [52]. In our study, the G57A mutation was identified in two isolates from Northern provinces in 2024, suggesting sustained circulation and geographical expansion of this mutation over time. In contrast, the G132A mutation appears to be novel, as it has not been previously reported in Vietnam. It also differs from findings in earlier studies from Latvia and China, where HAD-negative genotype II strains were associated with single-nucleotide deletions, short deletions, or point mutations such as G131A, G178A, G242A, and C301T [23,24]. The identification of distinct, independent mutations converging on disruption of *EP402R* suggests that loss of CD2v function, and consequently the HAD phenotype, may represent a recurrent evolutionary pathway. This convergence may reflect selective pressures in the field, potentially related to host immune responses or adaptation to new ecological or management conditions, although the biological consequences require further investigation.

Beyond *EP402R*, substantial genomic variation was observed in multigene family (MGF) regions, including MGF110, MGF360, MGF505, and deletions in MGF100. MGFs are known to play key roles in modulating host innate immune responses, particularly through antagonism of type I interferon signaling, as well as contributing to viral replication efficiency and virulence [53,54,55,56]. The accumulation of frameshift mutations and deletions in these regions, together with *EP402R* disruption, provides a plausible genomic basis for the attenuated phenotype and prolonged clinical presentation observed in infected pigs. However, ASFV genotype–phenotype relationships are complex, and definitive conclusions regarding virulence require controlled in vivo pathogenesis studies and functional assays. Analysis of the intergenic region (IGR) between *I73R* and *I329L* further demonstrated the presence of both IGR I and IGR II variants among the isolates. Previous studies have reported the predominance of IGR II in Vietnam [57,58]; however, our findings indicate co-circulation of multiple IGR variants, suggesting that ASFV evolution in Vietnam is not driven by a single dominant lineage but rather involves multiple co-existing lineages. This genetic heterogeneity may have implications for transmission dynamics and complicate disease control efforts.

The high genomic similarity observed between VNUA/ASFV/TB2024-isolate2 and VNUA/ASFV/HY2024-isolate3, despite being collected from different provinces, may indicate a recent common source or epidemiological linkage. Although detailed tracing data were unavailable, the close genetic relationship between these isolates could reflect regional dissemination of a closely related ASFV lineage through interconnected pig movement or production systems.

A particularly important finding of this study was the identification of VNUA/ASFV/VP2023-isolate1, which clustered phylogenetically with the *I177L*-deleted vaccine strain (ASFV-G-Δ*I177L*) licensed in Vietnam [17]. Comparative genomic analysis showed high nucleotide identity with ASFV-G-Δ*I177L* (99.6%) but clear differences from ASFV-G-Δ*I177L*/ΔLVR, primarily due to the absence of large MGF deletions [48]. Importantly, the previously reported ASFV-G-∆I177L/∆*EP402R* DIVA vaccine candidate did not harbor the *EP402R* point mutations identified here, as *EP402R* disruption in that virus was achieved through homologous recombination using a transfer vector containing a GFP gene under the control of the p72 promoter [19].

The presence of a 112-nucleotide deletion within the I177L gene, together with insertion of an mCherry cassette under the control of the p72 promoter, strongly suggests that this isolate originated from the ASFV-G-ΔI177L vaccine strain and subsequently acquired additional genetic changes during circulation under field conditions. In Vietnam, two vaccines based on live attenuated ASFV strains have been licensed since 2023 [18,59]. The detection of an ASFV-G-ΔI177L vaccine-like strain in the present study is consistent with previous reports describing the detection of ASFV-G-∆MGF vaccine-like strains in the field associated with chronic disease in finishing pigs and reproductive disorders in sows [60,61]. In addition, in vivo experimental studies have demonstrated that pigs vaccinated with ASFV-G-∆MGF strains may transiently shed vaccine virus, transmit the virus to contact pigs, and, in some cases, induce clinical disease in exposed animals [62].

These findings highlight the importance of continuous genomic surveillance and post-vaccination monitoring to better understand the evolution, transmission dynamics, and epidemiological risks associated with the use of live attenuated ASF vaccines under endemic field conditions. Importantly, our findings provide evidence suggesting post-release evolution of a vaccine-derived ASFV strain, raising critical considerations regarding biosafety, vaccine monitoring, and regulatory oversight. The acquisition of additional genomic alterations, including nonsense mutations in the *EP402R* gene and mutations within MGF regions, further supports the possibility that vaccine-derived ASFV strains may continue to evolve following field circulation.

A limitation of the present study is the absence of animal infection experiments. Therefore, future in vivo studies will be necessary to determine the pathogenicity, transmissibility, and clinical significance of these non-hemadsorbing ASFV strains. In addition, broader epidemiological investigations involving larger sample sizes and wider geographic surveillance will be important to determine whether similar vaccine-derived or recombinant ASFV strains are circulating in other regions of Vietnam.

In conclusion, this study provides important insights into the ongoing evolution of ASFV genotype II in Vietnam. Specifically, we document (i) the emergence of HAD-negative genotype II isolates associated with novel nonsense mutations in the *EP402R* gene, (ii) extensive genomic alterations in MGF regions potentially associated with attenuated phenotypes, (iii) co-circulation of multiple IGR variants, and (iv) the detection of an ASFV-G-Δ*I177L* vaccine-derived strain that underwent further evolution under field conditions. These findings highlight the dynamic nature of ASFV evolution and underscore the urgent need for continuous genomic surveillance, functional characterization of emerging variants, and careful monitoring of live attenuated vaccine deployment to ensure effective and safe ASF control strategies.

## Figures and Tables

**Table 1:** Whole genome sequencing characteristics of ASFV strains identified in this study.

Isolate	Collection Date	Original Sample	Location	Type of Pigs	Genome Length (nt)	Number of Reads	Number of ASFV Mapped Reads ^a^	Average Read Length (nt)	% of ASFV Mapped Reads	Mean of Depth Coverage ^b^	GC Content (%)	GenBank Accession No.
VNUA/ASFV/VP2023-isolate1	13 April 2023	Whole Blood	Vinh Phuc	Finishing pig	186,729	45,092,104	459,956	150	1.02	369×	38.6	PZ179805
VNUA/ASFV/TB2024-isolate2	13 May 2024	Whole Blood	Thai Binh	Sow	183,798	46,955,384	282,150	150	0.60	230×	38.5	PZ179806
VNUA/ASFV/HY2024-isolate3	6 July 2024	Whole Blood	Hung Yen	Finishing pig	183,745	33,979,598	103,533	150	0.30	85×	38.5	PZ179807

^a^ Mapped reads to the assembled sequence. ^b^ Sequencing depth was calculated using the following formula: average read length × number of reads matching the reference/reference genome size.

**Table 2:** ORFs with mutations detected in the three ASFV isolates *.

Gene	VNUA/ASFV/VP2023-Isolate1	VNUA/ASFV/TB2024-Isolate2	VNUA/ASFV/HY2024-Isolate3
*MGF 110-1L*	FM	NM	NM
*MGF 110-7L*	-	MD	MD
*ASFV G ACD 00190*	-	FM	FM
*MGF 110-10L—MGF 110-14L* fusion	FM	FM	FM
*ASFV G ACD 00240*	FM	-	-
*MGF 110-12L*	FM	-	-
*MGF 110-13Lb*	FM	FM	FM
*ASFV G ACD 00350*	FM	FM	FM
*MGF 360-10L*	-	FM	FM
*MGF 360-14L*	-	FM	FM
*MGF 505-2R*	-	FM	FM
*MGF 505-4R*	-	-	FM
*MGF 505-6R* and *MGF 505-7R*	-	MD	MD
*A238L*	-	FM	FM
*EP153R*	FM	FM	FM
*EP402R*	NM	NM	NM
*I177L*	MD	-	-
*MGF 100-3L*	MD	-	-
*I9R*	-	FM	FM

* Note: MD: Major Deletions; FM: Frameshift Mutations; NM: Nonsense Mutations; “-” indicates no mutations observed.

## Data Availability

The whole genome sequences of the three ASFV isolates generated in this study have been deposited in the NCBI GenBank database under the accession numbers PZ179805, PZ179806, and PZ179807. The raw sequencing data have been deposited in the NCBI Sequence Read Archive (SRA) under BioProject accession number PRJNA1467025.
